# The rehabilitation challenges of profound unilateral hearing loss

**DOI:** 10.1016/j.bjorl.2021.09.011

**Published:** 2022-03-05

**Authors:** Vagner Antonio Rodrigues da Silva, Arthur Menino Castilho

**Affiliations:** Universidade Estadual de Campinas (Unicamp), Faculdade de Ciências Médicas (FCM), Departamento de Otorrinolaringologia e Cirurgia de Cabeça e Pescoço, Campinas, SP, Brazil

Unilateral sensorineural hearing loss affects approximately 9% of the population and is less related to aging than bilateral hearing loss. Prevalence ranges from 0.3 to 1 per 1,000 newborns.^1^ Around 3–6% of school-aged children have some degree of unilateral hearing loss. Severe-to-profound unilateral hearing loss impairs sound localization, speech understanding in noisy environments (squelch effect), and leads to loss of the binaural summation effect.^2^

Patients with profound unilateral hearing loss or “SSD” (Single-Sided Deafness) have sufficient hearing for communication but have difficulties in sound localization and speech intelligibility in environments with competing noise. Child development is impaired, because it can cause delay in speech acquisition, poor school performance in up to 59% of the children, and behavioral changes.^3^

The majority of children with SSD are not rehabilitated. When the diagnosis is made, the school must provide special assistance, placing the child in the first rows, with the normal ear facing the teacher. In some cases, speech therapy should be used when language delay is present. In recent years, the possibilities for treating unilateral hearing loss have increased. In addition to the personal sound amplification device for partial losses and CROS (Contralateral Routing of Signal) systems, there are other treatment options for hearing rehabilitation on the affected side in cases of profound hearing loss such as Bone Anchored Hearing Aids (BAHAs) and cochlear implants (CI).

The CROS system and BAHAs effectively address the head shadow effect and restore sound perception to the non-listening side. However, they do not restore bilateral auditory input, which is necessary for true binaural hearing. BAHA users have a tendency for the satisfaction and benefit to decrease over time. Desmet et al.^4^ reported a 14% discontinuation over a 50 month period, about 3% per year. Another study showed cessation of use in approximately 30% of patients after 10 years. It is important to establish expectations and preconceptions of a CROS device prior to the surgery, particularly in patients with SSD. The difference in the abandonment rate over time may be due to developed perceptions that could be changed in the preoperative counseling. This counseling (habits, work/social environment, expectations) and an extensive preoperative testing of a BAHA in a band (headband or softband) can help patients figure their expectations. The choice of the best BAHA should be determined by the physician, who will discuss the clinical advantages and disadvantages of the devices with the patient after having collected all the qualitative and quantitative data derived from a complete audiological evaluation.

A large proportion of cases of children with congenital SSD are associated with structural abnormalities of the cochlear nerve or the VIII cranial nerve. Clemmens et al.^3^ described unilateral cochlear nerve (UCN) deficiency as a common cause of SSD. Nuclear magnetic resonance and computed tomography are indispensable tools in the preoperative assessment of the patient’s suitability for CI surgery, especially to rule out auditory nerve hypoplasia/aplasia, as an intact cochlear nerve is necessary for successful auditory rehabilitation after CI surgery. Thus, not all children with SSD may be suitable for cochlear implants. There is a critical period for the establishment of binaural integration in children. Younger age at the time of implantation is associated with better performance.

It is important to emphasize that many children with SSD can have an absolutely normal life and language development. The cause is usually congenital and, over the years, the child manages to adapt to this condition. For the treatment of these children, one must consider the actual benefit of using a device (implantable or not). As the patient and family sometimes have no complaints, this patient probably won’t use the proposed prosthesis. Although most of these devices are becoming more and more aesthetically adequate, their use in school can stigmatize the child or adult and make their daily use more difficult.

## Conflicts of interest

The authors declare no conflicts of interest.
